# Alcohol Consumption and Measures of Sarcopenic Muscle Risk: Cross-Sectional and Prospective Associations Within the UK Biobank Study

**DOI:** 10.1007/s00223-023-01081-4

**Published:** 2023-05-25

**Authors:** Jane Skinner, Lee Shepstone, Mary Hickson, Ailsa A. Welch

**Affiliations:** 1 Norwich Medical School, University of East Anglia, Norwich, NR4 7TJ UK; 2 School of Health Professions, University of Plymouth, Plymouth, PL4 8AA UK

**Keywords:** Alcohol, Muscle mass, Muscle strength, Cross-sectional analysis, Sarcopenia

## Abstract

Alcohol intake is a major modifiable risk factor for many diseases. Alcohol can also damage skeletal muscle health during ageing which in turn increases risk of sarcopenia, frailty and falls but this relationship is understudied. The aim of this study was to model the relationship between a full range of alcohol consumption and components of sarcopenic risk, skeletal muscle mass and function, in middle-aged and younger older-aged men and women. A cross-sectional analyses was undertaken of 196,561 white participants from the UK Biobank with longitudinal analysis also in 12,298 of these participants, with outcome measures for the latter repeated after around four years. For the cross-sectional analysis fractional polynomial curves were fitted in models of measures of skeletal muscle mass, appendicular lean mass/body mass index (ALM/BMI), fat-free mass as a percentage of body weight (FFM%) and grip strength, all predicted from alcohol consumption with models fitted for men and women separately. Alcohol consumption at baseline was based on the mean of up to five dietary recalls, typically over 16 months. Linear regression was used for longitudinal analyses to model the effects of alcohol consumption groups on these measures. All models were adjusted for covariates. In the cross-sectional analysis, modelled values of the muscle mass measures all showed a peak at medium levels of alcohol consumption and a steep decline with increasing alcohol consumption. Modelled differences in muscle mass from zero consumption of alcohol to 160 g/d ranged from 3.6 to 4.9% for ALM/BMI for men and women, respectively, and 3.6 to 6.1% for FFM%. Grip strength consistently increased with alcohol consumption. No association between alcohol consumption and muscle measures were seen in the longitudinal results. Our results suggest that higher levels of alcohol consumption could have detrimental effects on muscle mass in middle- and older-aged men and women.

## Introduction

The increased risk from alcohol consumption on cirrhosis of the liver, injuries, and liver, colorectal, breast and upper aerodigestive tract cancers is well-established [1]. In England in 2018/9, an estimated 358,000 hospital admissions had alcohol as the primary reason with risk of admission increasing by 6% than the previous year [2]. Estimates of the total economic costs attributable to alcohol were 2.5% of gross domestic product (GDP) in 2007 [3], equivalent to £54 billion in the UK in 2019. People in England aged 55 to 64 had the highest proportions drinking over the recommended limit: 38% of men and 19% of women [2].

Sarcopenia, the loss of skeletal muscle mass and function that occurs with increasing age [4], is associated with lower bone density, osteoporotic fractures [5], falls, frailty, hospitalisation and mortality [6]. Sarcopenia is highly prevalent in older populations (≥ 50 years): 1–29% in the community and 14–33% in long-term care [7]. Excess healthcare expenditures of $860 and $933 were estimated in the US in 2000 for every sarcopenic man or woman [8]. Therefore, prevention of the loss of skeletal muscle mass and function that leads to sarcopenia is important.

Mechanisms exist for alcohol to impact on the loss of muscle mass and strength during ageing with the potential impact increasing in those of older age, and at higher levels of consumption [9, 10]. The relationship between alcohol consumption and muscle mass and function, as risk factors for sarcopenia, has not been widely studied for higher levels of exposure [11–17] in large-scale general populations of both men and women. We hypothesised that higher levels of alcohol consumption would be associated with lower measures of skeletal muscle mass and grip strength.

This study examined cross-sectional and longitudinal associations between alcohol consumption and measures of sarcopenic risk, muscle mass and grip strength in a large cohort of middle-aged and younger older-aged men and women from the UK Biobank who have a wide range of alcohol consumption, including participants with high levels of intake.

## Methods

### Study Population

The UK Biobank is a prospective cohort study comprising 502,655 men and women aged 37–73 years, assessed from 2006 to 2010 in 22 assessment centres around the UK. The North West Multi-Centre Research Ethics Committee granted ethical approval for the study and all subjects gave written informed consent. Additional details of the rationale, design and survey methods for UK Biobank are available elsewhere [18].

### Participants in This Study

**Fig. 1:**
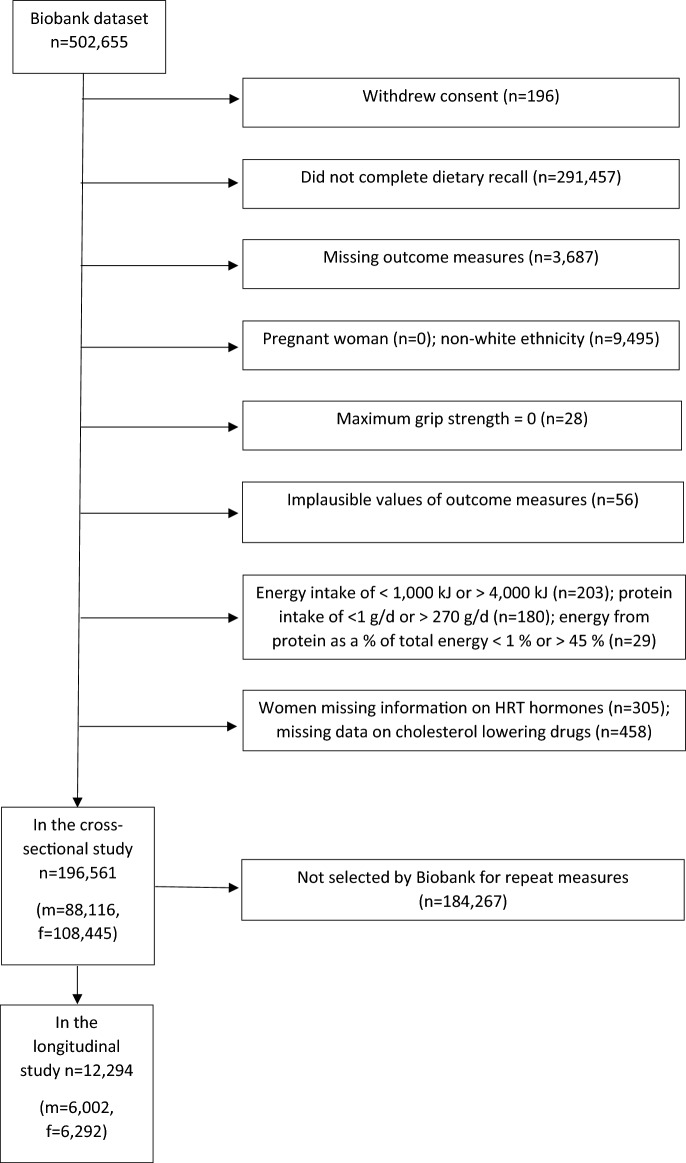
Selection of study participants

There were 502,459 people in the Biobank dataset after those who had withdrawn consent were deleted. We excluded (Fig. 1): participants without dietary questionnaires or measures of muscle mass and strength, those of non-white ethnicity, participants who had a grip strength of zero, those with extremes of the outcome measures or changes in these, or of energy or protein intake. We also excluded those with missing data on cholesterol-lowering drugs and women missing information on HRT. After these exclusions, 196,561 participants were used in the cross-sectional study. Within the Biobank dataset, a further 12,294 of these participants had the necessary data to be used in the longitudinal study.

### Measurements of Body Composition, Skeletal Muscle Mass and Grip Strength (Outcome Measures)

A Jamar J00105 hydraulic hand dynamometer was used to measure handgrip strength in both hands. In analysis, we took the higher of the two measurements.

A Seca 202 height measure was used to determine standing height without shoes. The Tanita BC 418MA was used to measure total body weight, following the removal of shoes and heavy clothing. Body mass index (BMI) was computed as weight (kg) divided by height (m) squared. The Tanita BC 418MA was also used to measure fat-free mass (FFM) with bioelectrical impedance. Appendicular lean mass (ALM) was calculated as the sum of lean mass in the arms and legs.

Since total FFM increases with body size we used the following accepted methods to scale for this:

- ALM divided by BMI (ALM/BMI), which was recommended by the FNIH Sarcopenia Project as its chosen measure of lean mass [19].
- FFM as a percentage of body weight (FFM%), defined as total FFM (in kg) divided by total body weight (in kg), multiplied by 100.

ALM/BMI, FFM% and grip strength were used as the outcome measures in our study.

A repeat assessment of 20,000 participants [18], including the outcome measures, was carried out an average of four years after the initial assessment. Subjects with both these repeat measurements and the baseline dietary questionnaires are included in the longitudinal analysis.

### Measurement of Alcohol Consumption and Other Dietary Intakes

The Oxford WebQ [20] alone was used to measure all dietary intakes used, including alcohol. This is an online self-reported 24 h recall questionnaire [20, 21] including 200 food items. A 24 h recall administered by an interviewer has been used to validate it [21]. Composition data from McCance and Widdowson’s *The Composition of Food* and its supplements [21] were used to calculate intakes of nutrients from this questionnaire. For the final 70,724 Biobank participants, the Oxford WebQ was included in baseline assessments and it was also made available on the web to all subjects with a known e-mail address (66% of the cohort) over a 16 months interval at four different time points [20]. The Oxford WebQ was therefore filled in by participants up to five times. As the first measurement was made at baseline for a subset of participants, the longest interval between questionnaires was 38 months, though it was usually smaller. Mean values of alcohol intake (g/day) were used for participants who completed the questionnaire more than once.

### Measurement of Potential Confounding Variables

Adjusted for in the analysis were age, smoking status (never, previous, current) and physical activity level (low, moderate, high or not known). The latter was derived from metabolic equivalents (METs) [22], which were calculated from questionnaire data. We used sex-specific quintiles of energy intake and percentage energy from protein intake, calculated using the mean of the available dietary questionnaire data. Also included were the number of dietary questionnaires completed, self-reported use of cholesterol-lowering medication (current yes or no) and, for women, hormone replacement therapy (HRT) use (current yes or no) and menopausal status (had menopause yes or no). Height was included in the grip strength models. All of these are well-established factors that impact on sarcopenic risk [23].

### Statistical Analysis

Participant characteristics were described by calculating means and SDs for continuous variables and proportion of participants for categorical variables by categories of alcohol intake (never drinker, former drinker and g/day categories of 0– < 1, ≥ 1– < 8, ≥ 8– < 16, ≥ 16– < 24, ≥ 24– < 32, ≥ 32– < 48, ≥ 48– < 80, ≥ 80). ALM/BMI, FFM% and grip strength were used as the outcome measures in our study. All analyses were stratified by sex, because of known differences in body composition between men and women.

In the cross-sectional analysis, fractional polynomial regression models were used to model the relationship between alcohol (g/day) as a continuous variable and measures of muscle mass and strength, departing from an assumption of linearity. We used a multivariable fractional polynomial model procedure developed by Royston and implemented as the *mfpa* command in Stata [24]. This command applies an algorithm to determine the best-fitting fractional polynomial of the predictor variable as a continuum. For specifics on this implementation of fractional polynomials and the default functions considered, see Royston and Sauerbrei [24]. An advantage of this method is it makes no prior assumptions about the nature of the relationship. Fitting quintiles of alcohol, for example, a categorisation often utilised in epidemiological analysis, can mean that detail is lost. It is possible to see this with our large sample size in the wide range of exposures observed; using quintiles, the highest quintile would consist mainly of participants with relatively low levels of alcohol consumption.

For the much smaller sample used for longitudinal change in outcomes, we used a linear model to assess the effects of alcohol exposure at baseline as a categorical variable on the second measurement of measures of muscle mass and strength, adjusting for baseline levels of the outcome, time between baseline and repeat measurements and baseline values of possible confounding variables.

The statistical analyses were carried out in Stata (version 17; Stata LP, College Station, Texas, USA).

### Sensitivity Analyses

We repeated the analyses for men and women aged < 65 and ≥ 65 separately, as sarcopenia is more prevalent in older age groups, since there is the potential that alcohol may interact with the greater losses of FFM that occur with increasing age. We also fitted models excluding those who said that they never drank or were former drinkers. Finally, we fitted models using FFM/BMI as an outcome. FFM/BMI is an established method of scaling and has been used in previous publications [25].

## Results

### Participant Characteristics

The cross-sectional analysis included 88,116 men and 108,445 women (Table 1). Estimation of alcohol intake and dietary covariates were based on recalls completed up to 38 months apart. Thirty-one percent of male participants and 41% of female participants consumed less than 1 g of alcohol per day. Fifty-one percent of male and 70% of female participants drank less than the UK Chief Medical Officer’s guidelines of 14 units/week (16 g/day). However, very high levels of alcohol consumption were also found, with 14% of men and 4% of women drinking ≥ 48 g/day.

**Table 1:** Characteristics and dietary intakes at baseline of study subjects aged 39 to 72 years

Men	Alcohol intake group (g/d) and (units/d)					
	Never drinker	Former drinker	0– < 1 0– < 1/8	≥ 1– < 8 ≥ 1/8– < 1	≥ 8– < 16 ≥ 1– < 2	≥ 16– < 24 ≥ 2– < 3
*n*	1090	2026	24,143	7194	10,664	11,012
Alcohol (g/day)	0.0 (0.0)	0.0 (0.0)	0.0 (0.1)	5.1 (1.8)	11.9 (2.3)	19.5 (2.1)
Alcohol (% total energy)	0.0 (0.0)	0.0 (0.0)	0.0 (0.0)	1.7 (0.8)	4.0 (1.3)	6.5 (1.9)
Age (years)	57.7 (8.2)	56.5 (7.9)	56.0 (8.3)	57.3 (7.9)	57.2 (7.9)	57.2 (7.9)
BMI (kg/m^2^)	27.8 (4.9)	27.9 (4.8)	27.7 (4.4)	26.9 (4.0)	26.9 (3.9)	27.1 (3.8)
ALM/BMI (m^2^)	1.038 (0.118)	1.041 (0.113)	1.051 (0.112)	1.060 (0.111)	1.061 (0.110)	1.058 (0.108)
Appendicular lean mass (ALM) (kg)	28.6 (4.5)	28.8 (4.4)	28.9 (4.2)	28.3 (3.8)	28.3 (3.8)	28.5 (3.7)
Fat-free mass (FFM) (%)	75.1 (6.5)	74.8 (6.5)	75.1 (6.1)	76.0 (5.9)	76.0 (5.8)	75.7 (5.6)
Hand-grip strength (kg)	40.5 (9.0)	40.3 (9.5)	41.9 (8.8)	42.0 (8.5)	42.2 (8.5)	42.4 (8.5)
Physical activity (MET/w)	33.7 (39.6)	35.3 (39.6)	35.4 (38.6)	32.2 (34.0)	31.5 (32.3)	32.8 (34.0)
Energy intake (kJ/d)	9117 (2761)	9382 (3275)	9104 (2940)	9217 (2379)	9309 (2379)	9499 (2527)
Dietary protein intake (g/d)	82.8 (26.3)	87.0 (30.9)	86.5 (29.0)	85.7 (22.2)	85.8 (23.0)	86.9 (24.9)
Dietary protein intake (% total energy)	15.7 (3.7)	16.1 (4.0)	16.5 (4.1)	16.1 (3.0)	15.9 (3.0)	15.7 (3.1)
No. of dietary questionnaires used	2.1 (1.2)	2.0 (1.2)	1.7 (1.0)	2.8 (1.1)	2.5 (1.2)	2.3 (1.2)
Smoking status						
Never (%)	83.2	41.3	55.9	62.4	57.6	52.3
Previous (%)	12.3	47.2	34.7	32.3	36.6	40.5
Current (%)	4.5	11.5	9.3	5.3	5.8	7.2
Cholesterol-lowering drug (%)	25.2	27.0	21.2	20.1	20.1	20.7

Men	Alcohol intake group (g/d) and (units/d)					
	≥ 24– < 32 ≥ 3– < 4	≥ 32– < 48 ≥ 4– < 6	≥ 48– < 80 ≥ 6– < 10	≥ 80 ≥ 10	All	
*n*	7409	11,957	9547	3074	88,116	
Alcohol (g/day)	27.7 (2.5)	38.8 (4.4)	61.0 (9.3)	102.3 (19.4)	22.1 (25.4)	
Alcohol (% total energy)	8.9 (2.4)	12.2 (3.5)	18.0 (5.2)	26.6 (7.5)	6.7 (7.5)	
Age (years)	57.4 (7.8)	57.2 (7.7)	56.7 (7.8)	55.3 (7.7)	56.8 (8.0)	
BMI (kg/m^2^)	27.1 (3.7)	27.5 (3.8)	27.8 (3.9)	28.4 (4.1)	27.4 (4.1)	
ALM/BMI (m^2^)	1.058 (0.110)	1.053 (0.107)	1.045 (0.104)	1.048 (0.105)	1.054 (0.109)	
Appendicular lean mass (ALM) (kg)	28.5 (3.7)	28.8 (3.8)	28.9 (3.8)	29.6 (4.0)	28.7 (3.9)	
Fat-free mass (FFM) (%)	75.6 (5.6)	75.1 (5.5)	74.6 (5.4)	74.0 (5.4)	75.3 (5.8)	
Hand-grip strength (kg)	42.5 (8.4)	42.4 (8.5)	42.4 (8.6)	42.5 (8.6)	42.2 (8.6)	
Physical activity (MET/w)	32.3 (33.0)	33.9 (34.9)	34.5 (36.2)	35.4 (39.5)	33.7 (35.8)	
Energy intake (kJ/d)	9655 (2407)	9946 (2583)	10,498 (2668)	11,894 (3103)	9603 (2745)	
Dietary protein intake (g/d)	87.2 (23.4)	88.1 (25.8)	89.4 (27.9)	93.8 (33.3)	87.2 (26.5)	
Dietary protein intake (% total energy)	15.5 (2.9)	15.2 (3.0)	14.5 (3.1)	13.4 (3.1)	15.7 (3.5)	
No. of dietary questionnaires used	2.5 (1.2)	2.2 (1.2)	2.1 (1.2)	1.8 (1.1)	2.2 (1.2)	
Smoking status						
Never (%)	48.9	43.1	37.1	33.4	51.1	
Previous (%)	43.3	47.0	48.9	47.8	39.9	
Current (%)	7.8	9.9	14.0	18.8	9.1	
Cholesterol-lowering drug (%)	20.2	22.8	23.0	23.3	21.5	

Women	Alcohol intake group (g/d) and (units/d)					
	Never drinker	Former drinker	0– < 1 0– < 1/8	≥ 1– < 8 ≥ 1/8– < 1	≥ 8– < 16 ≥ 1– < 2	≥ 16– < 24 ≥ 2– < 3
*n*	3295	2845	37,972	14,831	17,026	12,206
Alcohol (g/day)	0.0 (0.0)	0.0 (0.0)	0.0 (0.1)	5.0 (1.9)	12.1 (2.4)	19.6 (2.4)
Alcohol (% total energy)	0.0 (0.0)	0.0 (0.0)	0.0 (0.0)	1.9 (0.9)	4.6 (1.5)	7.3 (2.1)
Age (years)	57.6 (8.0)	56.3 (7.6)	55.6 (8.0)	56.2 (7.7)	56.2 (7.7)	55.8 (7.7)
BMI (kg/m^2^)	27.5 (5.9)	27.8 (6.2)	27.2 (5.4)	26.1 (4.8)	25.8 (4.5)	25.8 (4.4)
ALM/BMI (m^2^)	0.727 (0.093)	0.732 (0.098)	0.738 (0.091)	0.755 (0.090)	0.758 (0.089)	0.760 (0.090)
Appendicular lean mass (ALM) (kg)	19.6 (2.9)	19.9 (3.0)	19.7 (2.7)	19.4 (2.4)	19.3 (2.3)	19.3 (2.2)
Fat-free mass (FFM) (%)	63.2 (7.5)	62.9 (7.9)	63.4 (7.1)	64.7 (6.8)	65.0 (6.7)	65.0 (6.6)
Hand-grip strength (kg)	24.2 (6.4)	24.0 (6.6)	25.3 (6.3)	25.7 (6.2)	25.8 (6.1)	26.0 (6.1)
Physical activity (MET/w)	33.6 (34.9)	32.9 (33.7)	31.9 (32.2)	30.9 (30.1)	31.7 (29.6)	31.0 (29.2)
Energy intake (kJ/d)	8047 (2609)	8078 (3031)	7942 (2504)	8087 (2008)	8231 (2099)	8399 (2086)
Dietary protein intake (g/d)	77.2 (25.7)	76.8 (28.3)	77.9 (24.5)	78.0 (19.5)	78.4 (20.8)	78.8 (21.0)
Dietary protein intake (% total energy)	16.6 (4.0)	16.6 (4.1)	17.1 (4.2)	16.6 (3.2)	16.4 (3.3)	16.2 (3.3)
No. of dietary questionnaires used	2.1 (1.2)	2.1 (1.2)	1.8 (1.1)	2.8 (1.1)	2.5 (1.2)	2.4 (1.2)
Smoking status						
Never (%)	82.2	49.6	63.9	66.9	62.2	55.9
Previous (%)	13.8	40.5	29.2	28.8	32.7	38.1
Current (%)	4.0	9.9	6.9	4.3	5.2	6.1
Cholesterol-lowering drug (%)	16.0	15.3	11.2	9.3	9.1	8.0
Hormone-replacement therapy (%)	6.3	9.1	7.0	7.3	7.5	8.2
Menopause (%)	77.3	75.8	70.1	72.7	71.9	70.5

Women	Alcohol intake group (g/d) and (units/d)					
	≥ 24– < 32 ≥ 3– < 4	≥ 32– < 48 ≥ 4– < 6	≥ 48– < 80 ≥ 6– < 10	≥ 80 ≥ 10	All	
*n*	7910	8116	3745	499	108,445	
Alcohol (g/day)	27.9 (2.7)	38.4 (4.8)	58.7 (8.6)	96.7 (16.0)	12.2 (16.1)	
Alcohol (% total energy)	10.1 (2.8)	13.5 (3.8)	19.3 (5.5)	27.5 (7.7)	4.3 (5.7)	
Age (years)	55.8 (7.6)	55.5 (7.6)	54.4 (7.6)	52.7 (7.6)	55.8 (7.8)	
BMI (kg/m^2^)	25.9 (4.3)	26.2 (4.4)	26.6 (4.5)	27.7 (5.4)	26.5 (5.0)	
ALM/BMI (m^2^)	0.759 (0.087)	0.756 (0.088)	0.752 (0.088)	0.748 (0.090)	0.749 (0.091)	
Appendicular lean mass (ALM) (kg)	19.4 (2.2)	19.5 (2.2)	19.7 (2.3)	20.3 (2.5)	19.5 (2.5)	
Fat-free mass (FFM) (%)	64.8 (6.5)	64.5 (6.5)	63.9 (6.5)	62.9 (6.8)	64.2 (6.9)	
Hand-grip strength (kg)	26.1 (6.2)	26.1 (6.1)	26.4 (6.1)	27.1 (6.5)	25.6 (6.2)	
Physical activity (MET/w)	31.5 (29.5)	31.3 (30.7)	29.9 (28.7)	31.0 (32.8)	31.5 (30.9)	
Energy intake (kJ/d)	8554 (2141)	8835 (2288)	9439 (2473)	10,996 (3151)	8242 (2349)	
Dietary protein intake (g/d)	79.1 (21.8)	79.9 (23.2)	82.2 (26.2)	86.7 (33.1)	78.5 (22.9)	
Dietary protein intake (% total energy)	15.9 (3.3)	15.5 (3.3)	14.9 (3.3)	13.4 (3.4)	16.5 (3.7)	
No. of dietary questionnaires used	2.3 (1.2)	2.1 (1.2)	1.9 (1.1)	1.5 (0.9)	2.2 (1.2)	
Smoking status						
Never (%)	50.3	44.1	41.0	37.5	59.9	
Previous (%)	42.4	46.2	46.1	41.5	33.4	
Current (%)	7.3	9.8	12.9	21.0	6.7	
Cholesterol-lowering drug (%)	8.6	8.5	8.7	8.8	10.0	
Hormone-replacement therapy (%)	8.6	9.3	8.3	8.2	7.6	
Menopause (%)	70.6	70.2	64.3	55.3	70.9	

Values are mean (SD) unless stated as %.

Participant characteristics varied by categories of alcohol consumption as shown in the descriptive Table 1 (presented as groups based on the mean intake in (g/d), with the equivalent in (units/d)). For men: those drinking most were the youngest and had the greatest BMI and appendicular lean mass; ALM/BMI and FFM% and were greatest in the mid-range of alcohol intake; grip strength was lowest in non-drinkers; physical activity, energy intake and smoking increased with alcohol consumption but percentage energy from protein decreased with alcohol consumption; former and never drinkers were more likely to take cholesterol-lowering drugs, and the rate of taking these drugs increased with increasing alcohol intake. Results for women showed similar patterns, except that: physical activity did not show a clear relationship with alcohol consumption, though it was higher for never and former drinkers; HRT use was highest in the upper ranges of alcohol consumption but the percentage of women having reached the menopause declined with increasing alcohol consumption.

### Cross-Sectional Measures of Muscle Mass and Strength

**Fig. 2:**
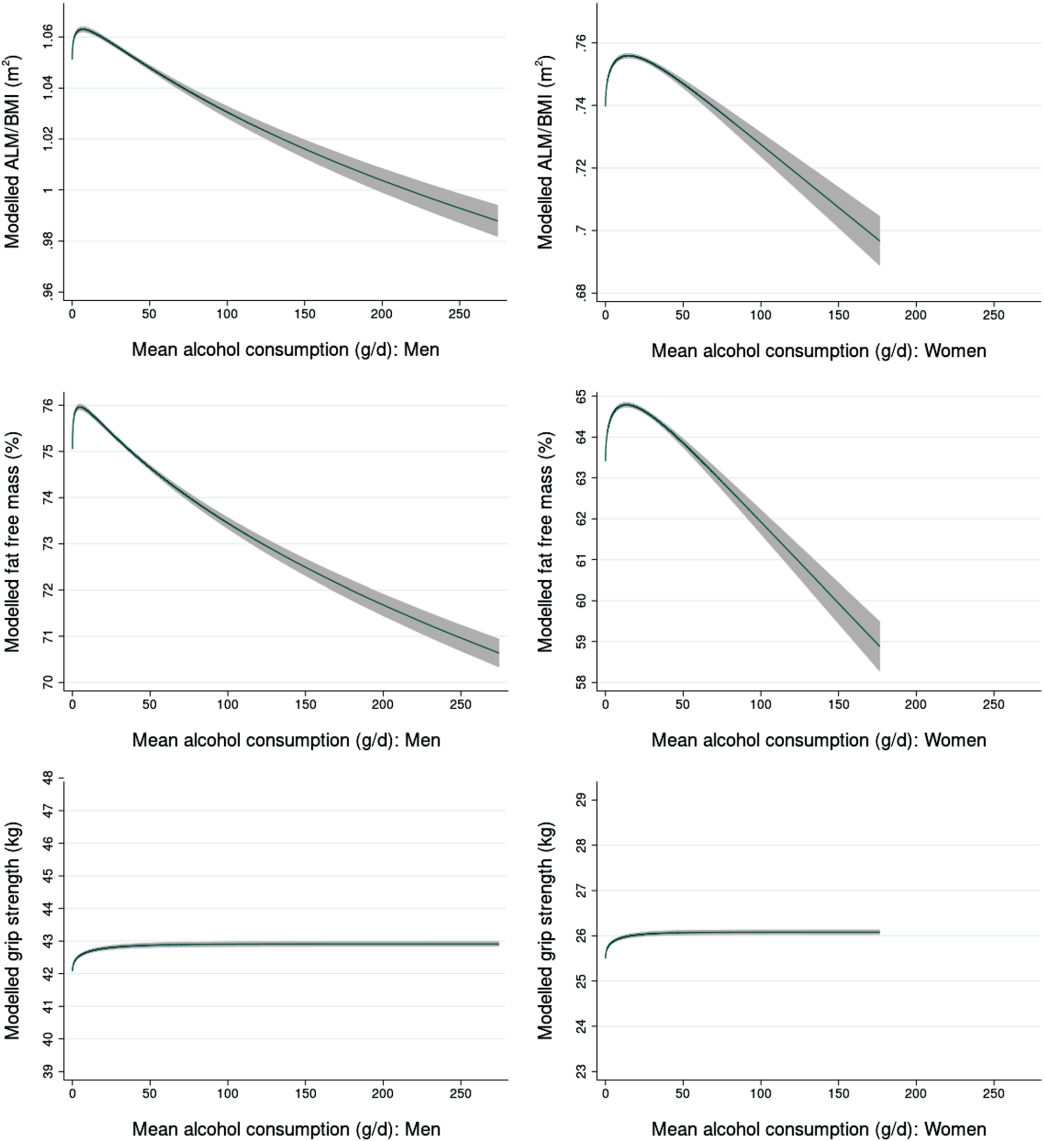
Fitted fractional polynomial curves modelling measures of muscle mass and strength by alcohol consumption. Adjusted for age, physical activity, energy intake, protein intake (% total energy), smoking status, no. of food recalls used, cholesterol-lowering drugs, HRT and menopausal status (women), height (grip strength only)

Figure 2 shows the modelled fractional polynomial curves for ALM/BMI, FFM% and grip strength, all predicted from alcohol consumption, for men and women separately. The graphs are scaled so that the Y-axis covers one standard deviation of the outcome variable. The fitted values for selected values of alcohol consumption are shown in Table 2. The other model variables were fixed to their average values.

**Table 2:** Modelled values (95% CI) of measures of muscle mass and strength for selected values of alcohol consumption

Alcohol (g/d)	Alcohol (units/d)	Modelled values								
		ALM/BMI (m^2^)	95% CI	% difference from 0	Fat-free mass (FFM) (%)	95% CI	% difference from 0	Hand-grip strength (kg)	95% CI	% difference from 0
Men										
0	0	1.051	(1.050, 1.052)		75.1	(75.0, 75.1)		42.1	(42.0, 42.2)	
2	¼	1.061	(1.060, 1.062)	0.93	75.9	(75.8, 75.9)	1.11	42.4	(42.4, 42.5)	0.84
4	½	1.063	(1.061, 1.064)	1.08	76.0	(75.9, 76.0)	1.21	42.5	(42.5, 42.6)	1.07
6	¾	1.063	(1.062, 1.064)	1.12	76.0	(75.9, 76.0)	1.20	42.6	(42.5, 42.6)	1.23
8	1	1.063	(1.062, 1.064)	1.12	75.9	(75.9, 76.0)	1.16	42.6	(42.6, 42.7)	1.33
12	1½	1.062	(1.061, 1.063)	1.05	75.8	(75.8, 75.9)	1.01	42.7	(42.6, 42.8)	1.49
16	2	1.061	(1.060, 1.062)	0.94	75.7	(75.6, 75.7)	0.85	42.7	(42.7, 42.8)	1.59
24	3	1.058	(1.057, 1.059)	0.66	75.4	(75.4, 75.5)	0.50	42.8	(42.7, 42.9)	1.72
32	4	1.055	(1.054, 1.056)	0.36	75.2	(75.1, 75.2)	0.16	42.8	(42.8, 42.9)	1.80
48	6	1.049	(1.048, 1.050)	− 0.23	74.7	(74.6, 74.8)	− 0.47	42.9	(42.8, 42.9)	1.89
80	10	1.037	(1.035, 1.039)	− 1.34	73.9	(73.8, 74.0)	− 1.55	42.9	(42.8, 43.0)	1.96
160	20	1.014	(1.010, 1.017)	− 3.59	72.3	(72.1, 72.5)	− 3.64	42.9	(42.8, 43.0)	1.99
Women										
0	0	0.740	(0.739, 0.741)		63.4	(63.3, 63.5)		25.5	(25.4, 25.6)	
2	¼	0.750	(0.749, 0.750)	1.34	64.3	(64.2, 64.3)	1.37	25.8	(25.7, 25.8)	1.09
4	½	0.752	(0.752, 0.753)	1.71	64.5	(64.5, 64.6)	1.75	25.9	(25.8, 25.9)	1.39
6	¾	0.754	(0.753, 0.755)	1.92	64.6	(64.6, 64.7)	1.95	25.9	(25.9, 25.9)	1.56
8	1	0.755	(0.754, 0.756)	2.05	64.7	(64.7, 64.8)	2.07	25.9	(25.9, 26.0)	1.69
12	1½	0.756	(0.755, 0.757)	2.17	64.8	(64.7, 64.8)	2.17	26.0	(25.9, 26.0)	1.86
16	2	0.756	(0.755, 0.757)	2.19	64.8	(64.7, 64.8)	2.16	26.0	(26.0, 26.0)	1.97
24	3	0.755	(0.754, 0.756)	2.05	64.7	(64.6, 64.7)	1.97	26.0	(26.0, 26.1)	2.10
32	4	0.753	(0.752, 0.754)	1.78	64.4	(64.4, 64.5)	1.64	26.0	(26.0, 26.1)	2.17
48	6	0.748	(0.746, 0.749)	1.08	63.9	(63.8, 64.0)	0.81	26.1	(26.0, 26.1)	2.24
80	10	0.735	(0.733, 0.738)	− 0.57	62.7	(62.5, 62.9)	− 1.10	26.1	(26.0, 26.1)	2.27
160	20	0.703	(0.696, 0.710)	− 4.92	59.5	(59.0, 60.1)	− 6.10	26.1	(26.0, 26.1)	2.28

Adjusted for age, physical activity, energy intake, protein intake (% total energy), smoking status, no. of food recalls used, cholesterol-lowering drugs, HRT and menopausal status (women), height (grip strength only)

ALM/BMI and FFM% fitted values show peaks at moderate levels of alcohol consumption for men (6.8 and 4.8 g/d, respectively) but the peaks are higher for women (14.7 and 13.5 g/day), though the maximum is quite flat. After the peaks, both sexes show a monotonic decline with increasing alcohol consumption. All these outcomes were lower for alcohol intakes above 48 g/day in men and 80 g/day in women compared to the zero-consumption category. The percentage difference for men is around 1.5% lower at 48 g/day, 1.5% at 80 g/day and 3–4% at 160 g/day. For women, it is 0.5–1% lower at 80 g/day and around 5% lower at 160 g/day.

Hand-grip strength increased for the range of alcohol intake, showing a higher difference at very low levels. The value at 160 g/day was roughly 2% higher than the value at zero alcohol consumption, for both men and women.

When the cross-sectional analysis was restricted to men and women aged < 65years (*n* = 165,261, 84% of the sample) the fractional polynomial models (not shown) were similar to the overall results. For those aged ≥ 65 (*n* = 32,300) the relationship was less clear, possibly due to the smaller sample size and small number of individuals in the high alcohol consumption categories. There was no difference to the models fitted when excluding those who said that they never drank or were former drinkers. Models of FFM/BMI produced curves that were almost identical to those for ALM/BMI.

### Longitudinal Measures of Muscle Mass and Strength

Length of follow-up varied from 2.1 to 6.1 years (mean 4.0, SD 0.8). Results of the longitudinal analyses are shown in Table 3. No association was seen between alcohol consumption at baseline and indices of muscle mass and strength at follow-up. For each model, the coefficients for the effect of alcohol consumption, compared to no consumption, were estimated to be small and were non-significant. There were small absolute numbers with very high levels of consumption, however (22 women and 170 men ≥ 80 g/day), which did not let us examine exposures that high or higher.

**Table 3:** Beta coefficients (95% CI) from linear regression of repeat measures of muscle mass and strength by alcohol consumption group

Baseline alcohol (g/d)	Baseline alcohol (units/w)	ALM/BMI (m^2^)				Fat-free mass % (FFM%)			Hand-grip strength (kg)		
		*n*	Beta	95% CI	*p*	Beta	95% CI	*p*	Beta	95% CI	*p*
Men					0.629			0.397			0.402
0– ≤ 16	0– ≤ 14	3035	Reference			Reference			Reference		
> 16– < 57.1	> 14– < 50	2444	− 0.0010	(− 0.0031, 0.0011)	0.359	− 0.0659	(− 0.2193, 0.0876)	0.400	0.0923	(− 0.2313, 0.4158)	0.576
≥ 57.1	≥ 50	523	0.0000	(− 0.0037, 0.0037)	0.987	− 0.1763	(− 0.4498, 0.0972)	0.206	0.3929	(− 0.1826, 0.9683)	0.181
Women					0.741			0.3479			0.833
0– ≤ 16	0– ≤ 14	4429	Reference			Reference			Reference		
> 16– < 40	> 14– < 35	1519	− 0.0002	(− 0.0022, 0.0018)	0.841	− 0.0659	(− 0.2534, 0.1215)	0.491	0.0768	(− 0.2055, 0.3592)	0.594
≥ 40	≥ 35	344	− 0.0015	(− 0.0053, 0.0023)	0.441	− 0.2489	(− 0.6044, 0.1066)	0.170	− 0.0529	(− 0.5875, 0.4817)	0.846

Adjusted for baseline levels of the outcome, time between baseline and repeat measurements, and baseline levels of age, physical activity, energy intake, protein intake (% total energy), smoking status, no. of food recalls used, cholesterol-lowering drugs, HRT and menopausal status (women), height (grip strength only)

## Discussion

### Summary

Our cross-sectional results from the UK Biobank Study showed that measures of skeletal muscle mass, ALM/BMI and FFM%, both increased with moderate alcohol consumption and then declined consistently and substantially with higher levels of consumption. However, the increase in muscle mass seen for low levels of consumption was much smaller than the decrease seen for high levels. The highest differences in indices of muscle mass for an intake of 80 g/day compared to one of zero were 4–6%. Grip strength increased with alcohol throughout the range of intakes, rising to an increase of around 2%. These results held for both men and women. The modelled percentage differences in ALM/BMI of 1.34% for men and 0.57% for women for an alcohol consumption of 80 g/day correspond to an absolute difference in ALM of 0.6 kg and 0.13 kg, respectively, for a BMI of 25. Excluding those who said that they never drank or were former drinkers made no difference to the models fitted. In the longitudinal analysis, no association was seen with alcohol consumption groups for any of the indices of muscle mass and strength. However, the number of individuals with follow-up data was 6% of those included in the cross-sectional analyses, which may have impacted on our findings.

### Mechanisms of Harm

Mechanical harm to skeletal muscle caused by alcohol involves decreased diameter of type II fibres, especially type IIb fibres (which contain few mitochondria), whilst the type I fibres appear unaffected, even exhibiting compensatory hypertrophy in the initial phase [10, 26]. Existing evidence shows that alcoholic myopathy can be sufficiently substantial to alter mid-arm circumference and muscle measured by DXA (Dual X-ray Emission Absorptiometry) [10, 26]. Regular excessive alcohol consumption (> 80 g alcohol/day) may cause chronic alcoholic myopathy, muscle weakness and wasting [10, 26]. Moreover, considerable muscle damage attributed to alcohol intake has been observed even in healthy subjects who drank 28 units daily for four weeks [10] and alcoholic myopathy is present in 40–60% of individuals with alcoholism [10]. Heavy alcohol consumption [27] has also been found (≥ 20 g/day for women) to attenuate the protective influence of protein intake against low skeletal muscle index development in women, but not in men (values ≥ 40 g/day considered). Processes implicated in alcoholic myopathy include: damage to membranes, decreased rates of protein synthesis, an increase in RNase activities, loss, breakdown and redistribution of ribosomal RNA, production of free radicals and modified Ca2+ regulation [10, 26]. Other mechanisms involved may include secretion of inflammatory cytokines and glucocorticoids [10]. Chronic excess alcohol consumption can lead to dysbiosis of the gut microbiota and autophagy-induced hyperammonaemia, instigating upregulation of muscle protein breakdown and downregulation of muscle protein synthesis through deactivation of IGF-1 and activation of myostatin, AMPK and REDD1 [9].

### Comparison with Other Studies

Prior research suggests that high ingestion of alcohol in individuals with alcoholism is an issue for skeletal muscle mass, but the full range of alcohol intake it has been less studied in general populations. An earlier meta-analysis found no evidence of an increased risk of sarcopenia with alcohol consumption [16, 28]. However, individuals were only categorised into drinkers or non-drinkers and so dose was not investigated. A recent meta-analysis, which looked at the highest vs. lowest alcohol consumption categories for the studies included found no association with sarcopenia overall, but an increased risk for those < 65 years in a subgroup analysis [28]. Several studies have examined muscle mass in a middle-aged or elderly population with a focus on alcohol intake but only one had more than 5000 participants [11], and all but two were cross-sectional [14, 15]. Furthermore, only four studies were able to examine the effect of very high alcohol intake: these measured intakes of ≥ 50 g/day [13], > 64 g/day [17], up to 68.9 g/day [29] and ≥ 80 g/day [12]. Within our study 14% of men and 4% of women drank equivalent to or more than 48 g alcohol per day, the equivalent of six units Three of the previous studies found results that concur with ours: lean mass (%) decreased with alcohol consumption [13] and heavy alcohol consumption was associated with a reduced skeletal muscle mass index [12] and an increase in grip strength with alcohol category in both sexes [29]. The other study found no association with higher levels of alcohol consumption and the relative appendicular skeletal muscle index [17]. Studies also did not adjust for protein intake [12, 13, 17, 29] or for physical activity [12]. A previous study on the cross-sectional Biobank data found no association between sarcopenia and alcohol consumption but used only tertiles of alcohol intake, so not taking into account the full upper range of alcohol consumption [30]. In comparison, our study investigated a large population of both men and women a large difference in alcohol consumption with both cross-sectional and longitudinal analyses full adjusted for known factors that contribute to differences in muscle mass and function in populations, particularly physical activity and protein consumption.

In our cross-sectional analysis, the peaks of the measures of muscle mass occurred at 7 g/day of alcohol for ALM/BMI for men and 5 g/day for FFM%, whereas the peaks for women were higher, both at around 14 g/d, respectively. Compared with the harms of alcohol and other chronic disease conditions these are lower than the nadir of risk of 31 g/day seen from continuous dose–response meta-analysis for ischaemic heart disease mortality in men but broadly similar to that of 11 g/day for women [31]. A previous two-stage dose–response meta-analysis [32] found that incident frailty risk decreased until consumption of 15 g/day and increased thereafter.

Grip strength in this study was found to increase with alcohol consumption in both men and women. This has been suggested previously, with one US study [11] finding that light and low-moderate alcohol consumption were significantly associated with increased grip strength compared to reference category (12 drinks/y) although this study also found an association between a history of sustained excessive drinking and lower grip strength. A significant increasing linear trend has also been seen with grip strength and alcohol consumption in Japanese men and women [29]. However, a further longitudinal investigation, found that alcohol consumption was associated with a decline in grip strength in adjusted models for both sexes [14]. Whilst we adjusted our analyses for physical activity, we acknowledge we may have incompletely adjusted for this aspect of our analyses. This especially applies to the type of physical activity, which was not available. There is increasing evidence that specifically resistance, strength and power training are beneficial for prevention of sarcopenia and grip strength loss [33].

### Scale of Findings in Comparison with Yearly Loss

Muscle mass and functional loss occurs with ageing (along with loss of strength is known as sarcopenia) [34]; the median rate of muscle mass decline is estimated as 0.47%/years for men and 0.37%/years for women. This compares to differences of around 5% for our measures of muscle mass seen in very high drinkers (> 160 g/day) compared to those who reported no alcohol consumption, suggesting an effect of very high alcohol equivalent to 10-13yrs of ageing.

Data from the English Longitudinal study of Aging [35] showed that grip strength declines at a rate of 0.5 kg/year in men and 0.3 kg/year in women aged 50 and over. The authors did not report mean grip strength by sex, but these correspond to losses of just over 1%/years, using mean grip strengths from our sample. This is in contrast to our data, where we found differences of around 2% seen for heavier drinkers in our results.

Actual losses for ALM/BMI and FFM% (as percentage per year) seen in the Biobank longitudinal sample for men were 0.7% and 0.3% and for women were 0.4 and 0.1%. Grip strength showed a loss as high as 3.7% per year for men and 4.7% per year for women, however. Although this is higher than shown in other studies, the reason is not clear.

### Further Strengths and Weaknesses of This Study

We believe that this study is the first to model measures of muscle mass and strength at the highest levels of alcohol consumption with adjustment for potential confounding and factors known to influence muscle health (e.g. protein intake). Measures of muscle mass are important due to the relationship with sarcopenia, frailty and falls. An advantage of our study is that we also used fractional polynominals to model the non-linear relationship found, rather than linear regression or other methods in which the nature of the relationship is assumed a priori. It contrasts with dividing the exposure into quantiles, where information would be lost for the higher exposures. The large sample size allowed us to investigate levels of alcohol consumption much greater than commonly defined as high, in contrast to the highest levels seen in other studies (≥ 50 g/day [13], > 64 g/day [17], up to 68.9 g/day [29] and ≥ 80 g/day [12]). Daily alcohol intake was also reported by participants using 24 h recall, which was averaged over up to five recalls, so may not be a particularly good measure of habitual intake for participants with one or two recalls. Participants may not remember their consumption accurately or report it correctly in 24 h dietary recalls, particularly for stigmatised intakes such as heavy drinking. This might lead to under-reporting of alcohol intake, but this is unlikely to be differentially related to muscle mass or function status. Another weakness is that we included in our analysis only white participants. This was due to differences in body composition between white and non-white participants and the small numbers of non-white participants, so that we were not able to analyse them separately. We cannot infer causation from our study, as the major parts of the analyses were cross-sectional, with this study design having a high potential for residual confounding. Also, the longitudinal elements of the sample were not significant. We acknowledge that these were based on a much smaller sample size with few very heavy drinkers, which may be why we were unable to find an effect. We do, however, have a large sample of the general population and we found these associations even in people of middle age which could have implications for those in older age.

## Conclusion

Our results suggest that alcohol may have detrimental effects on muscle mass at higher levels of consumption in middle- and older-aged people. Further data are required to confirm these findings and understand the inconsistencies in the results found between muscle mass and strength. Nevertheless, these data suggest another reason to avoid high habitual consumption of alcohol in middle and early older age.
